# Parent-Child Interaction Therapy in a Case of Global Developmental Delay and Leukoencephalopathy

**DOI:** 10.3389/fpsyt.2018.00427

**Published:** 2018-09-10

**Authors:** Reem M.A. Shafi, Jennifer L. Vande Voort, Paul E. Croarkin, Magdalena Romanowicz

**Affiliations:** Department of Psychiatry and Psychology, Mayo Clinic, Rochester, MN, United States

**Keywords:** psychotherapy, developmental delay, behavioral issues, novel modifications, language development

## Abstract

Parent-child interaction therapy (PCIT) is an evidence-based, behavioral dyadic treatment for caregivers and their children aged 2–7 years old with emotional and behavioral disorders. Here we present a treatment course of a 3-years-old girl with leukoencephalopathy, dysgenesis of the brainstem, and associated global developmental delay who was diagnosed with muscular dystrophy after PCIT completion. At the beginning of PCIT she had the developmental level of an 18 months old with language skills of a 12–18 months old; both her vocabulary and verbal expression were very limited. She had slow, unco-ordinated gait with limited fine motor skills. She was referred to Psychiatry with concerns regarding disruptive behaviors including severe self-injury. PCIT was started with a focus on PRIDE skills (Praise, Reflection, Imitation, behavioral Description and Enjoyment); particularly behavioral description and reflection with simple developmentally appropriate labeled praise. Modifications to treatment included using non-verbal actions (e.g., “high fives” as praises), sign language and using only one-step basic commands, which greatly improved compliance. In a matter of weeks, the patient demonstrated remarkable improvement in her disruptive behavior as evidenced by parent/daycare report and clinical observation. Surprisingly her vocabulary more than doubled and her ability of self-expression also increased; she was able to point to things and ask for them. This clinical experience suggests that PCIT principles are effective treatment interventions for other clinical presentations outside of the usual inclusion criteria. Implementation of targeted PCIT interventions greatly benefited the development of language skills and communication in a young child with global developmental delay.

## Background

Parent-child interaction therapy (PCIT) is an evidence-based dyadic behavioral treatment for caregivers and their children between 2 and 7 years old with emotional and disruptive behavior disorders (1). PCIT is divided into two phases: child directed interaction (CDI) and parent directed interaction (PDI). The overarching focus of both is to positively reframe the caregiver-child relationship to optimize a secure relationship utilizing operant conditioning techniques and play therapy (1). CDI focuses on positive interactions between caregiver and child while PDI focuses on caregiver ability to give and follow-through on commands.

Traditional PCIT was developed for treatment of disruptive behavior disorders in developmentally normal children. However, various adaptations have shown its benefit for both internalizing and externalizing psychological disorders, behavioral manifestations of autism, and intellectual disability in 3–6 years old (2–4).

This case report presents the treatment course of a very young child with global developmental delay with the aim of demonstrating how modified PCIT appeared to increase her vocabulary, self-expression, and behavior.

### Demographics, prenatal and neonatal period

The patient was a 3-years-old Caucasian female (developmental age estimated at 18 months) with no psychiatric history. She was born at 39 weeks gestation via C-section with Apgar scores of 8 and 9 at 1 and 5 min, respectively. Her birth weight was 4.07 kg, length 51.5 cm and head circumference 34.5 cm. She had a normal Minnesota newborn screen. She developed normally for 4–5 months until visual, gross and fine motor, language and cognitive concerns emerged resulting in referrals to Neurology and Ophthalmology. Unremarkable family history noted.

### Specialist assessments: pediatric neurology and ophthalmology

Neurological assessment at 5 months indicated central hypotonia with increased peripheral tone. An EEG was ordered, due to intermittent unresponsiveness, which yielded normal results with no evident seizure activity. At 6 months she was noted to have difficulty with visual tracking and inward orientated gaze bilaterally. She was diagnosed with amblyopia, mypoia and early onset esotropia that required surgical correction at 10 months.

She was neurologically reassessed at 12 months. In an effort to establish an etiology, several tests were performed including chromosomal microarray, peroxisomal disorder testing, lysosomal disorder testing, mucopolysaccharide test, creatine phosphokinase test, thyroid function tests, ammonia, and lactate; all results were within normal limits. Genetic testing for 100 genes of leukoencephalopathy was completed with no positive result. When she was 4 years of age whole exome testing was performed and she was found to be a compound heterozygote for mutations in the POMGNT1 gene, which can cause a spectrum of phenotypes including muscular dystrophy. A diagnosis of muscular dystrophy was subsequently confirmed upon muscle biopsy. Two mutations in the POMGNT1 gene were identified: c.1539 + 1G > A inherited from mother and c.932G > A inherited from father (hence a compound heterozygote). Mutations in the POMGNT1 gene cause POMGNT1-related disorders, an autosomal recessive spectrum of phenotypes. Our patient was found to be moderately affected, with a phenotype similar to what is seen in type B3 (muscle-eye-brain disease) (5). This condition includes eye findings (myopia, strabismus and ocular atrophy), brain findings (white matter changes, cerebellar hypoplasia) with delayed motor development and learning disability, as well as muscular dystrophy (5).

Brain MRI showed white matter paucity and abnormal signal in periventricular regions consistent with periventricular leukomalacia. She was diagnosed with leukoencephalopathy with brainstem dysgenesis. Serial MRI scans did not show progression or improvement.

She continued to have neurology, ophthalmology, pediatrics and genetics follow up after the completion of PCIT.

### Development

At 5 months, she was able to fix her gaze on her parents but was limited in her interactions. At 7 months, she was able to sit independently, scoot, army crawl and vocalize noises but not syllables. At 15 months, she had obvious gross motor delay with inability to stand or crawl. At this point in time, on the Ireton Developmental Scale, she was at the level of a 10-months-old child. At 16 months, she continued to be limited to army crawling and rolling on the floor. She was babbling primarily with vowels but had one or two non-specific vowel-consonant combinations. She was able to clap and high five but not wave. She was not following one-step commands with gesture or directing her gaze to specifically named people. She has been at/below 2nd percentile for weight throughout her life. At 21 months, she was still not walking or standing independently.

### Psychiatry referral

At 3.5 years, she was referred to psychiatry for worsening disruptive behavior, including severe self-injury and daily head-banging that occasionally required a helmet. At times she would lower herself to the ground, while screaming and head-banging. Several times a week when angry, she would seek self-injury and subsequently look for attention in an attempt to communicate her frustration. The disruptive behavior occurred at home, school, and daycare culminating in an episode of fracturing a teacher's nose.

It was noted that the patient began to have behavioral problems at 2.5 years when her parents divorced. She subsequently lived at home with her mother and 13-years-old sister although remained in frequent contact with her father with whom she had a good relationship. However, her mother did report that there was significantly less structure in the father's home and usually there was more behavioral issues immediately after the patient would come back from the father's home. On thorough psychiatric assessment and review of the electronic medical record there was no concern for past or present occurrence of trauma or abuse of the patient.

Prior to seeing psychiatry she had already received extensive multidisciplinary treatment including occupational and speech therapy with limited results. It was hypothesized that some of the behaviors were related to struggles with self-expression.

### PCIT intervention

At the beginning of PCIT treatment, the patient was able to walk with no assistance, although her gait was slow and uncoordinated. She would frequently fall and was not able to run. A short walk to the office often necessitated a break or required her Mother to carry her. In terms of her fine motor skills she was able to draw a line and scribbles. Her grasp was fisted grip and she could hold a pencil for only brief periods. She could say approximately three words (“mama, up, hey”) inconsistently and use a limited number of signs. Her receptive language skills were in the 12–18 months range. PCIT was started with the main focus being on PRIDE skills, particularly behavioral description and reflection with simple, developmentally appropriate, labeled praise (“do skills”). Modifications to treatment included: using non-verbal actions (e.g., “high fives” as praises), sign language, and using only one-step commands (e.g., “hand me this cup” while pointing to the cup), which greatly improved compliance. Mother was encouraged to reflect everything the patient said, including all the sounds that were reasonable and could be spelled out, such as “yay” and “ouch” (Table 1).

**Table 1:** Table of PCIT modifications.

**Session number**	**Protocol**	**Modification**
CDI 1–4	Verbal labeled praise	Non-verbal actions for labeled praise including “high fives.”
CDI 1–4	Reflect words	Reflect all reasonable sounds.
CDI 1–4	Behavioral description	Mother used future tense “you will build with blocks” to help organize the play.
CDI 1–4	Enthusiasm	Even a small gain (a sound made by patient) was met with significant enthusiasm by mother.
PDI 1–3	Verbal commands	Sign language. Simple gestures such as pointing while giving a command. Every attempt at compliance was praised.
PDI 1–3	Time out routine	Time out corner instead of room was used. Not left alone during time out. Mom stood with her back facing patient while patient stood in the corner

**Figure 1:**
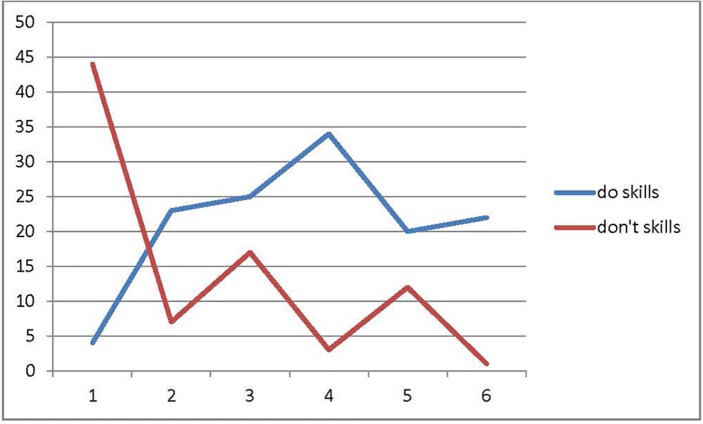
Results of parenting skills (observational) based on DPICS coding during first 5 min of appointments.“do skills” are behavior descriptions, reflections, labeled praises; “don't skills” are questions, commands and negative talk.

The first four PCIT sessions focused on CDI coaching with Mother who attended all the sessions without another caregiver. On initial assessment, Mother used many questions and commands without praise. The patient was able to comply initially but quickly lost interest and would wander off when she appeared unsure of tasks. With utilization of the “bug-in-the-ear” system and live coaching, her Mother was able to increase labeled praise for desired behaviors, and she focused more on tasks and followed Mother's lead. Parenting skills were measured using the Dyadic Parent-Child Interaction Coding System (DPICS) (6) during all appointments, except the eighth (graduation session) and sixth (data missing). Please see Figure 1 that outlines an increase in Mother's “do skills” (increased from 4 to 22) and a decrease in her “don't skills” (decreased from 44 to 1).

In her third session, the patient presented with increased speech while Mother reported gain of two new words and ability to point. Mother reached mastery on labeled praise (used 10 skills in 5 min time) and behavior description (used 12 skills in 5 min time). She also reflected 90% of what her daughter said. Mother also reported her daughter's ability to engage in special playtime for 5 min daily with no behavioral concerns. Patient was able to demonstrate incremental increases in focusing on tasks in each session, from staying on task for approximately 5 s during first appointment to at least 3–5 min toward treatment end.

**Figure 2:**
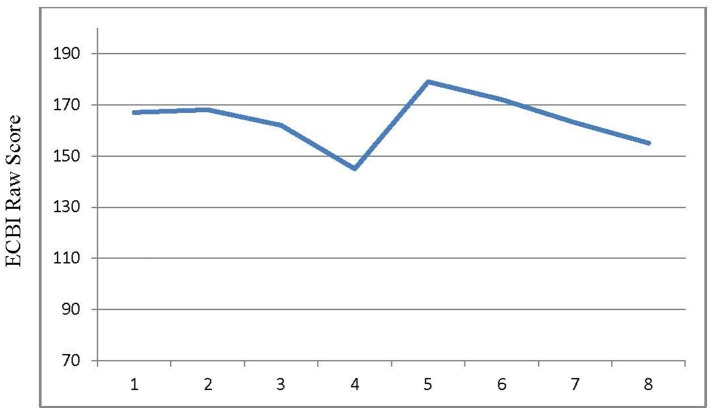
Results for externalizing behavior problems (parent report). ECBI (Eyberg Child Behavior Inventory) clinical cut-off score is 132; x-axis is the session number.

Initially, the PDI component (which included the time-out procedure) proved challenging. As shown in Figure 2, the patient's Eyberg Child Behavior Inventory (ECBI) scores (7) increased when Mother started making more demands on her. However, with coaching, the patient was able to grasp the concept of her non-compliance with consequences for her behavior. Due to the direct observation and live coaching, it was determined that she did best with one-step commands. This observation was of great benefit, as Mother was able to act accordingly and relayed this information to daycare.

ECBI scores were obtained at each session, except for baseline. Her overall total scores did not change and went from 167 on session 1 to 155 on session 8 (Figure 2). However, when evaluating particular items on the questionnaire, such as dawdling, getting angry, having difficulty self-entertaining or being alone, she went from 7 to 5 or 4. Some items, such as having a short attention span, being easily distracted, or failing to finish projects, remained high at 7; however, that may be a reflection of her developmental level of an 18-months-old child. A subsequent neurological evaluation reflected broad improvement. She had improved behavior in all settings and had attainment of eight words and five signs, which was attributed to PCIT. Her self-injurious behavior decreased in frequency and severity from almost daily to once a week, per Mother's report. Patient was able to point to things and communicate needs more effectively like asking for food or drink. She had no difficulty riding the bus, attending school or complying with self-care (she would wear her glasses when asked). Despite total ECBI score remaining in the clinically significant range, her therapist and Mother decided to stop PCIT after 8 sessions completed over 3 months due to clinically significant improvement.

The treatment team has been communicating with the patient's Mother due to the confirmed diagnosis of muscular dystrophy. Palliative care is now involved and the team wanted to offer support. Mother mentioned that the patient has been doing very well in school and at home. She has been listening and following directions to her capabilities. She is even able to take a special school bus with no difficulties. Patient's vocabulary has also continued to grow, which can be attributed to her overall development, ongoing speech therapy, and PCIT. It is important to recognize that prior to PCIT, the patient was receiving intensive speech therapy with little progress, and it was only after PCIT was initiated that the patient started making significant gains in her speech.

## Discussion

To our knowledge, this is the first case report of a child with significant developmental delay (estimated age of 18 months), multiple medical issues, and externalizing problems for whom PCIT was an effective treatment. During assessment, we hypothesized that the patient had emotional and behavioral difficulties partially due to her inability to express herself. Her language skills were so limited that it was exceedingly difficult to assess her expressive-receptive abilities. She also had not responded to intensive speech therapy as expected; partially due to her defiant behavior and refusal to participate. Based on previous studies, there are clear connections between developmental delay, behavioral issues and communication difficulty (8, 9).

Prior evidence suggests when a parent follows their child's lead, language improvement can occur for children with developmental delays (9). A study by Garcia et al. showed that PCIT had a mediating effect on child behavioral problems via improvement of language production (10). Study participants were children between 20 and 70 months, who were either at risk for or had mild developmental delay. The authors noticed that similar to previous studies (11), child-directed interactions in the presence of their caregivers who were using “do skills” (i.e., PRIDE skills) led to a significant growth in the child's language skills.

Of note is that the patient's progress was not reflected on ECBI, which may be explained partially by the fact that ECBI is mostly used to track children who develop normally. For example, our patient continued to wet the bed and continued to need help with a number of things which gave her high scores on ECBI; however, that was not necessarily in the context of her defiance. Therefore, ECBI may not be the best standard objective measure for this particular patient. Clinical observations, along with parent report, paired with the patient's school performance were the best way to capture the patient's progress in treatment. It is interesting that in this case, she demonstrated improvement in disruptive behavior both at home and daycare, in a matter of weeks. Surprisingly, her vocabulary more than doubled and her ability for self-expression also increased. Her behavioral improvement was noted by the treating neurologist and family to be secondary to PCIT. This may be partially explained by parent-child interaction improvement that led to decreased defiance and frustration, which facilitated learning.

## Conclusion

Targeted PCIT implementation greatly improved language development skills and communication in a young child with global development delay. PCIT principles are effective treatment interventions for other clinical presentations outside of the usual inclusion criteria.

## Ethics statement

We have obtained verbal informed consent from the patient's guardian and the data has been anonymized to assure confidentiality.

## Author contributions

MR and RS drafted the initial manuscript, reviewed and revised the manuscript. JV and PC critically reviewed and revised the manuscript. All authors approved the final manuscript as submitted and agree to be accountable for all aspects of the work.

### Conflict of interest statement

The authors declare that the research was conducted in the absence of any commercial or financial relationships that could be construed as a potential conflict of interest.

## Abbreviations

PCIT: parent child interaction therapy
CDI: child directed interaction
PDI: parent directed interaction
EEG: electroencephalogram
CPK: creatine phosphokinase
MRI: magnetic resonance imaging
ECBI: Eyberg Child Behavior Inventory
PRIDE, praise, reflection, imitation: behavioral description and enjoyment.
